# Patient–Registered Nurse Communication: Exploring Patients' Expectations and Experience in Primary Care Consultations

**DOI:** 10.1111/hex.70353

**Published:** 2025-07-22

**Authors:** Sofia Östensson, Mariela Acuña Mora, Laura Darcy, Lotta Saarnio Huttu, Sandra Van Dulmen, Annelie J. Sundler

**Affiliations:** ^1^ Faculty of Caring Science, Work Life and Social Welfare University of Borås Borås Sweden; ^2^ Nivel (Netherlands Institute for Health Services Research) Utrecht the Netherlands; ^3^ Department of Primary and Community Care, Radboud Institute for Health Sciences Radboud University Medical Center Nijmegen the Netherlands

**Keywords:** communication, expectations, experiences, patient, primary care, registered nurse, satisfaction

## Abstract

**Introduction:**

Primary care is the first point of contact for patients in most Western healthcare systems, addressing acute, chronic and preventive health needs. Registered nurse‐led first visits (new episode) consultations have shown promise in improving accessibility and meeting the growing demand for timely care. Understanding how patients evaluate their interactions with registered nurses is a crucial first step towards identifying communication needs and promoting person‐centred care. Therefore, the aim of this study was to explore patients' expectations and experiences with registered nurse communication in primary care consultations.

**Methods:**

A prospective cohort study was conducted. The QUOTE‐COMM (Quality Of communication Through the patient's Eyes) questionnaire, with three scales (affect‐oriented, task‐oriented and therapy‐oriented), was completed by patients before and after RN‐led consultations to assess their expectations and experiences.

**Results:**

A total of 138 patients participated in the study. Patients reported high expectations and positive experiences regarding registered nurses' communication during consultations. The highest scores were observed on the affect‐oriented scale. No significant differences were found between patient expectations and experiences in relation to patient age, sex and educational level. However, patients whose native language was not Swedish rated the affect‐oriented scale less positively. Additionally, longer consultations were associated with higher satisfaction at 2 weeks follow‐up.

**Discussion:**

This study highlights patients' high expectations and positive experiences in connection with communication in registered nurse consultations. The findings underscore the need for further research into various aspects of communication with patients in primary care, aiming to better understand and enhance the quality of care in this setting.

**Conclusion:**

Patients appear to have high expectations in connection with registered nurse communication in primary care, and it seems that registered nurses overall succeed in meeting these expectations.

**Patient or Public Contribution:**

The study was conducted within Swedish primary care, where patient and public involvement in research is considered essential. Registered nurses assisted with patient recruitment, and a total of 138 patients shared their experiences with researchers, and the results have been discussed with a reference group comprising representatives of patients and registered nurses.

**Clinical Trial Registration:**

ClinicalTrials.gov identifier: NCT06067672.

**Abbreviations**
B: Beta
IQR: Interquartile range
*n*
: number of participants
RN: Registered Nurses
*p*
: *p* value
SD: standard deviation
*z*
: z‐test, the mean of the distribution

## 1 Introduction

Primary care is the first point of contact with the healthcare system in most Western countries and plays a central role in managing acute, chronic and preventive health concerns. Ensuring timely, appropriate and cost‐effective access to primary care presents significant challenges. Registered nurse‐led consultations have emerged as a strategy to improve accessibility and availability, addressing the growing demand for timely care. Although there is growing international interest in understanding the contributions and roles of RNs in primary care settings [1], the expanding role of RN‐led consultations in triage and patient management remains underexplored. Further investigation is needed to address this gap. Appropriate patient management is highly dependent on the quality of the nurse–patient communication. Examining communication during these consultations can therefore give valuable insights into the dynamics of the interaction and the effectiveness of the communication strategies used [2].

Primary care is a complex field, within which patients differ in terms of their health status, ethnic background, socio‐economic status and age [3]. RNs in primary care work across the lifespan, meeting different age groups and building short‐ and long‐term relationships with patients. They manage a wide range of tasks, from addressing acute life‐threatening conditions to providing wound care, vaccinations and chronic disease management. To adequately perform these tasks, RNs must possess strong communication skills to ensure accurate information is delivered and to promote patients' autonomy and active participation in their care [4, 5].

Communication is central in all RN–patient interactions [6] and represents a complex process that is essential for understanding patients' health conditions [7]. Effective communication plays a critical role in identifying and addressing patients' needs and health concerns [8]. High‐quality communication can lead to better health outcomes, improved adherence to treatments and health advice, and greater patient satisfaction [7, 9]. While the primary goal of medical communication is to support patient health, its impact can be observed across various outcomes—immediate, immediate mediate and more long‐term [2]. Moreover, various factors—such as the RN's and the patient's age and sex—can influence the communication process [10, 11]. To ensure optimal care, healthcare providers need to tailor their communication to individual patient needs [12].

Deficiencies in communication skills can negatively affect patient care and outcomes. Research highlights the critical role communication plays in patient satisfaction, with poor communication negatively influencing the quality of care and patient health [9, 13]. Moreover, the initial impression appears to significantly shape patient experiences and satisfaction with care, and as such influences both the content and outcomes of RN–patient encounters. Simple acts of courtesy, both verbal and nonverbal, such as smiling, introducing oneself and demonstrating attentiveness, are considered essential by patients Ekman et al. [14]. Patient satisfaction is an important indicator of healthcare quality, which is influenced by patients' expectations and experience of care. Patients expect to be taken seriously and for professionals to be competent, empathetic and attentive to their individual needs [15]. Comparing patients' expectations with their experiences has been identified as a crucial factor for gaining a comprehensive understanding of patient–clinician consultations and satisfaction [16].

Understanding how patients experience their interactions with RNs is an initial step towards identifying their needs and promoting person‐centred communication. Increased understanding of these interactions can support the development of effective communication strategies in RN‐led primary care consultations. The QUOTE‐COMM instrument has proven to be both feasible and useful in measuring patients' communication needs and experiences, as well as for identifying areas for improvement [17]. It can provide insight into patients' expectations and experiences of their communication with RNs and highlight the importance of various aspects of that communication. Similar to the work by van Weert et al. [18], this study used two indicators of person‐centred communication that are *importance*, for example, how important a specific aspect is to the patient, and *performance*, for example, the patient's experience of that aspect. These are indicators of what patients consider important in healthcare [18]. Previous research using the QUOTE‐COMM has explored patients' expectations and experiences with communication in general practitioner (GP) consultations [19], cancer care [17] and age‐related macular degeneration [20]. These studies conclude that communication is essential for meeting patients' needs and preferences, as well as reducing anxiety and treatment burden. Despite existing knowledge, there remains a lack of research on patients' communication needs and preferences in primary care consultations with RNs. Therefore, the aim of this study was to explore patients' expectations and experiences with RN communication in primary care consultations. Three research questions were addressed:

- What are patients' expectations and experiences of communication when consulting with RNs?
- How satisfied are patients with the RN consulting experience?

Does the patient's age, sex, native language, educational level and length of consultation have any association with their expectations and experiences?

## 2 Methods

A prospective cohort study based on questionnaire data was conducted.

### 2.1 Sample and Setting

In this study, a consecutive sampling strategy was used to target patients seeking primary care. Data were collected at primary care centres in southwestern Sweden. All RNs at the participating primary care centres were approached with a request to collaborate in the study. All patients who visited participating RNs due to a new health problem or a new episode of a current health problem during pre‐selected study days were invited to participate. Inclusion criteria were patients aged 18 years or older. All participants provided written informed consent.

Data were collected at 10 primary care centres representing diverse locations and clinic sizes, including both public (*n* = 8) and private (*n* = 2) facilities in rural and urban areas. These centres served between 5000 and 12,000 patients. This study is part of the PINPOINT project [21] that, based on a sample size calculation, aimed at recruiting 150 patients.

There are approximately 1200 primary care centres in Sweden, operated by both public and private providers. All primary care is funded through a tax‐based healthcare system that ensures coverage for everyone living or working in the country, and care is generally consistent regardless of the provider's ownership [22]. Approximately 40.5 million visits are made annually to primary care facilities, with more than 12 million of these involving RNs. Generally, the RNs' responsibilities range from management of acute and chronic conditions to providing broader support [8]. While most RN visits are assumed to focus on chronic disease management, the proportion of same‐day, RN‐led consultations for new health problems or new episodes of existing conditions remain unknown. Traditionally, these types of consultations have been the primary responsibility of GPs within the Swedish primary care system.

### 2.2 Data Collection

The data reported by patients was collected by means of a questionnaire at three distinct time points:

- The first data collection (T0) was conducted before the consultation to assess patients' expectations.
- The second data collection (T1) took place immediately after the consultation to capture patients' immediate experiences.
- The third data collection (T2) was conducted 2 weeks after the consultation to follow up on patients' experience.

The data collection was undertaken between September 2023 and March 2024. Out of 152 eligible patients in the PINPOINT project, 138 patients answered the questionnaire (response rate 95%). Eligible patients who did not participate were mainly unable to do so due to time restraints.

### 2.3 Instrument

The instrument used for measuring the participants' expectations and experiences in relation to various aspects of communication was the Quality Of communication Through the patient's Eyes (QUOTE‐COMM) [17, 23] and has been demonstrated to be feasible and useful [17]. The QUOTE‐COMM instrument measures various aspects of communication that have been identified as valuable to patients in terms of both their expectations and their experiences. It contains three scales: a task‐oriented scale that assesses different medical content (six items), an affect‐oriented scale that evaluates the emotional aspects of the interaction (seven items) and a therapy‐oriented scale that measures the therapeutic aspects of the consultation (six items). In total, QUOTE‐COMM consists of 19 items reported on four‐point Likert scale (1 = not important/no, 2 = fairly important/not really, 3 = important/on the whole, yes and 4 = extremely important/yes). The internal consistency of the questionnaire was assessed using Cronbach's *α*. For the task‐oriented scale, values were *a* = 0.74 (T0), *a* = 0.82 (T1) and *a* = 0.85 (T2). For the affect‐oriented scale, values were *a* = 0.45 (T0), *a* = 0.56 (T1) and *a* = 0.69 (T2). For the therapy‐oriented scale, values were *a* = 0.77 (T0), *a* = 0.74 (T1) and *a* = 0.69 (T2).

Participants also answered a short questionnaire that contained demographic questions on year of birth, sex, native language and level of education.

### 2.4 Data Analysis

The analysis was conducted using the Statistical Package for the Social Sciences (SPSS), version 28.

Demographic data is reported with descriptive statistics (i.e., percentages, frequencies, mean, standard deviation, median and interquartile range). Due to the non‐normal distribution of the data, non‐parametric tests were used, and medians and interquartile ranges were reported. Patient satisfaction was determined through Wilcoxon signed‐rank tests, by comparing patient expectations versus immediate experiences (T0 vs. T1), and expectations versus 2 weeks follow‐up (T0 and T2).

Multivariate linear regression analyses were conducted to examine associations between patients' age, sex, native language, educational level and consultation length with their expectations (T0), immediate experiences (T1) and patients' experience at 2 weeks follow‐up (T2) related to the RN consultation. Age, length of consultation and education level were dichotomised for analysis. Age was categorised as under 65 years (*n* = 85) and 65 years or older (*n* = 53). Consultation length was categorised as either less than 10 min (*n* = 81) or 10 min and longer (*n* = 52). Educational level was categorised in primary or secondary education (99 participants) and higher education (39 participants). All tests were two‐sided and the significance level was established at *p* < 0.05.

### 2.5 Ethical Considerations

Ethical approval was obtained from the Swedish Ethical Review Authority (Dnr Ö24‐2023/3.1). Participant sampling, data storage and access followed relevant laws and safety regulations to ensure participants' security, privacy and confidentiality. All participants received detailed information about the study, including the right to refuse, data processing and the right to access their personal data.

## 3 Results

### 3.1 Demographic Characteristics

Sex distribution among the 138 participating patients was balanced between women (*n* = 75, 54.3%) and men (*n* = 63, 45.7%). Their ages ranged between 18 and 90 (mean age 55.02, SD 19.36). The majority of the participants reported Swedish as their native language (*n* = 116, 84.1%), while a smaller proportion had another native language (*n* = 22, 15.9%). More than half had primary or secondary education (*n* = 81, 58.7%) or higher education (*n* = 55, 41.3%). The average duration of the consultation was 10.34 min (SD = 5.75).

### 3.2 Patients' Expectations of Communication With RNs

Patients reported high expectations (T0) before RN consultations across all three communication scales assessed: task‐oriented (median 3.51), affect‐oriented (median 3.8) and therapy‐oriented (median 3.3)—see Table 1.

**Table 1:** Overview of the median, IQR and Wilcoxon signed‐rank tests of patients' expectations, experiences and 2 weeks follow‐up.

Items	Pre‐consultation (Expectations T0) (*n* = 138)	Post‐consultation (Immediate experiences T1) (*n* = 135)	Follow‐up (2 weeks follow‐up T2) (*n* = 112)
	Median (IQR)	Median (IQR)	Median (IQR)
Task‐oriented aspects	3.51 (0.84)	3.66 (0.83)	3.50 (0.83)*
Examines me/The RN examined me	4 (0.01)	4 (1.0)	4 (1.0)
Diagnoses what's wrong/The RN diagnosed what's wrong	4 (1.0)	4 (1.0)	4 (1.0)
Explains well what's wrong/The RN explained well what's wrong	4 (1.0)	4 (1.0)	4 (1.0)
Gives advice on what to do/The RN gave advice on what to do	4 (1.0)	4 (1.0)	4 (1.0)
Helps me with my problem/The RN helped me with my problem	4 (0.0)	4 (1.0)	4 (1.0)
Informs me well about the treatment/The RN informed me well about the treatment	4 (1.0)	4 (1.0)	4 (1.0)
Affect‐oriented aspects	3.80 (0.71)	4.00 (0.14)*	3.86 (0.57)
Is friendly/The RN was friendly	4 (1.0)	4 (0)	4 (0)
Takes my problem seriously/The RN took my problem seriously	4 (0)	4 (0)	4 (0.50)
Listens to me well/The RN listened to me well	4 (0)	4 (0)	4 (1.0)
Is open to me/The RN was open to me	4 (0)	4 (0)	4 (1.0)
Takes enough time for me/The RN took enough time for me	4 (1.0)	4 (0)	4 (1.0)
Is empathic to me/The RN was empathic to me	4 (1.0)	4 (0)	4 (1.0)
Gives me enough attention/The RN gave me enough attention	4 (0.01)	4 (0)	4 (1.0)
Therapy‐oriented aspects	3.3 (0.68)	2.8 (1.5)*	3.0 (1.34)*
Takes the final decision about the treatment/The RN took the final decision about the treatment	3 (2.0)	4 (1.0)	3 (2.0)
Prescribes medication/The RN prescribed medication	3 (2.0)	3 (3.0)	3 (3.0)
Discusses different treatment options/The RN discussed different treatment options	3 (1.0)	3 (3.0)	3 (2.0)
Referrers me to another specialist/The RN refereed me to another specialist	3 (1.0)	4 (2.0)	3 (2.0)
Involves me in making a decision/The RN involved me in making a decision	4 (1.0)	4 (1.0)	4 (1.0)
Informs me about side effects/The RN informed me about side effects	4 (1.0)	3 (3.0)	3 (2.0)

* Significant difference between T0 versus T1 and T0 versus T2.

### 3.3 Patients' Experiences of Communication With RNs

Overall, patients also reported their experiences with RNs' communication during consultations as high, both immediately after the consultation and at 2 weeks follow‐up. Immediately after the consultation, (T1) patients reported the highest scores on the task‐ and affect‐oriented scales. When comparing expectations before (T0) and experiences immediately after (T1) consultations, patients reported significantly higher scores on the affect‐oriented scale (*z = *−3.174, *N*‐Ties *=* 119, *p* < 0.005) after the consultation.

Patients reported their experience at the 2 weeks follow‐up (T2) was also high, see Table 1. When comparing total mean scores for patients' expectations (T0) with their experiences at the 2 weeks follow‐up (T2), there was no significant difference (*z* = −0.320, *N*‐Ties *=* 103, *p* = 0.748). For the difference scale, a significant difference was found on the task‐oriented scale (*z* = −3.53, *N*‐Ties *=* 96, *p* < 0.001), with lower median scores at the 2 weeks follow‐up (T2), compared to expectations (T0). On the therapy‐oriented scale, the results indicated significantly lower scores (*z* = −3.319, *N*‐Ties *=* 67, *p* < 0.001) for treatment‐related aspects of communication when comparing expectations (T0) with immediate experiences (T1) after the consultations, and this difference related to lower scorings remained at 2 weeks follow‐up (T2) (*z* = −3.42, *N*‐Ties *=* 65, *p* < 0.001).

### 3.4 Associations Between Patient Characteristics and Their Expectations and Experiences

No significant differences were found in relation to patients' age, sex and education when comparing their expectations (T0) with immediate experiences (T1) of the RN communication. Patients' native language was associated with lower scores on the affect‐oriented scale after the consultation (T1) (B = −218, *p* < 0.005)—see Table 2.

**Table 2:** Multivariate analyses of potential correlates of communications aspects.

	Expectations (T0)			Immediate experiences (T1)			Follow‐up (T2)		
	Task‐oriented aspect	Affect‐oriented aspect	Therapy‐oriented aspect	Task‐oriented aspect	Affect‐oriented aspect	Therapy‐oriented aspect	Task‐oriented aspect	Affect‐oriented aspect	Therapy‐oriented aspect
Correlates	B (SE) ᵝ	B (SE) ᵝ	B (SE) ᵝ	B (SE) ᵝ	B (SE) ᵝ	B (SE) ᵝ	B (SE) ᵝ	B (SE) ᵝ	B (SE) ᵝ
Age									
Under 65 years,	−0.15 (0.09)	−0.05 (0.13)	−0.01 (0.12)	0.07 (0.13)	−0.03 (0.07)	0.24 (0.24)	−0.72 (0.11)	−0.10 (0.78)	0.01 (0.19)
65 years or older	−0.14	−0.03	−0.01	0.06	−0.04	0.13	−0.55	−0.12	0.01
Sex									
Women,	0.28 (0.09)	0.74 (0.13)	0.16 (0.12)	0.90 (0.13)	0.15 (0.08)	0.05 (0.28)	0.12 (0.11)	0.08 (0.07)	0.20 (0.20)
Men	0.27	0.08	0.13	0.07	0.19	0.03	0.97	0.10	0.11
Native language									
Swedish, other	0.25 (0.12)	−0.17 (0.18)	−0.17 (0.16)	−0.10 (0.17)	−0.21 (0.10)	0.07 (0.28)	0.00 (0.16)	−0.17 (0.10)	−0.15 (0.24)
Native language	0.01	0.08	−0.10	−0.05	0.19	0.13	−0.00	−0.14	−0.06
Length of consultation									
Less than 10 min,	0.21 (0.97)	−0.11 (0.13)	0.19 (0.12)	0.18 (0.13)	−0.01 (0.08)	0.24 (0.24)	0.31 (0.11)	0.30 (0.07)	0.26 (0.20)
10 min and longer	0.20	−0.08	0.01	0.14	−0.13	0.13	0.24	0.36	0.15
Education									
Secondary school,	−0.20 (0.95)	−0.11 (0.13)	−0.33 (0.12)	−0.05 (0.13)	−0.07 (0.07)	−0.18 (0.22)	−0.00 (0.00)	−0.00 (0.00)	−0.69 (0.06)
University degree	−0.19	0.08	−0.10	−0.05	−0.09	−0.10	−0.04	−0.00	−0.12

Level of significance: < 0.05; < 0.001.

**Figure 1:**
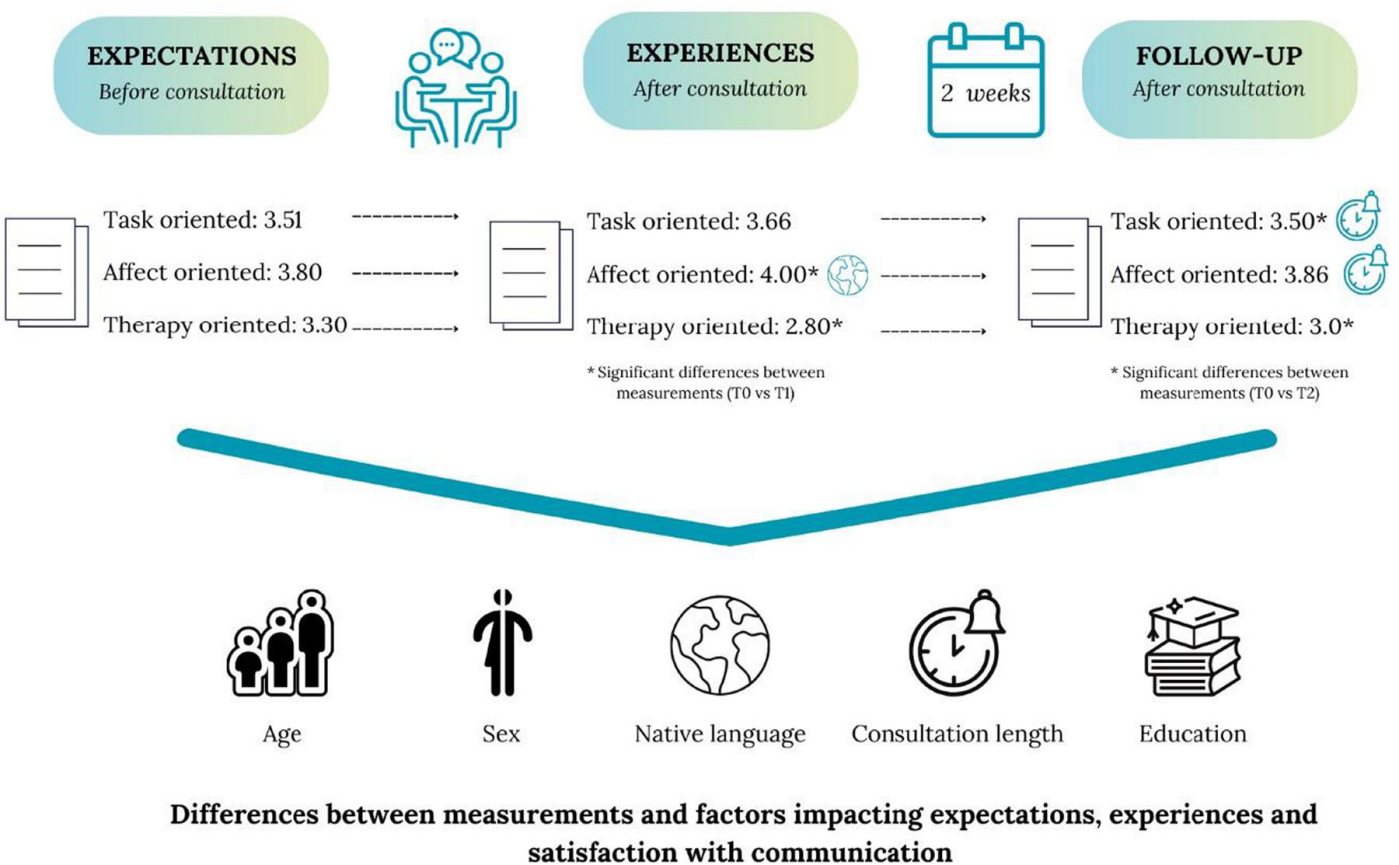
Overview of results.

When comparing expectations (T0) with 2 weeks follow‐up (T2), the results indicate a significant association between the length of the consultation and the task‐oriented scale (B = 0.31, *p* < 0.005) as well as the affect‐oriented scale (B = 0.30, *p* < 0.001). Participants with a longer consultation, lasting 10 min or more, reported higher levels of satisfaction with RN communication during the consultation, see Figure 1.

## 4 Discussion

This study found that patients had high expectations, which were generally met by RNs, both in their immediate experience (T1) and at follow‐up (T2). The overall high levels of expectations and experiences regarding communication suggest that interactions with RNs were highly valued and considered important by patients. Moreover, it indicates that RNs' communication was person‐centred, as their performance, as experienced by patients, was generally consistent with what patients considered important. Patient satisfaction is central to quality care and is strongly influenced by patients' experience with healthcare professionals' communication [15]. A recent review points to RNs' communication as central in fostering meaningful and effective communication [6]. Moreover, high‐quality communication has been shown to improve patients' adherence to treatment plans and medical advice, enhance health outcomes and increase overall satisfaction with care [7, 9]. This underscores the pivotal role of communication in meeting patients' expectations and in delivering person‐centred healthcare.

Although overall experience levels were high, differences were observed at 2 weeks follow‐up (T2) regarding patients' satisfaction with the RNs' communication on the task‐oriented scale. Significant differences were also found on the therapy‐oriented scale in patient experience (T1 and T2) when compared with their expectations (T0). The highest scores were observed on the affect‐oriented scale in all three measurements. This indicates the importance of interpersonal aspects in patient encounters and suggests that patients perceived the nurses as friendly, attentive, open and serious about addressing their concerns. These attributes are also foundational to person‐centred care, as they foster a sense of safety, security and trust while enhancing patient confidence [24]. This highlights the critical role of emotional communication in RN consultations. Navigating symptoms and emotional concerns during illness involves addressing emotional experiences such as fear, vulnerability and anxiety. Focusing on the emotional aspects of care promotes more empathetic and comforting communication [25]. Moreover, emotional communication can promote coping mechanisms and enhance overall well‐being [26, 27, 28].

Post‐consultation immediate experience levels (T1) were slightly higher, mirroring results from Barrat and Thomas [29], where patients with high expectations of RN competence who perceived the use of advanced practical skills tended to report greater satisfaction after consultations. Similarly, patients rated RN communication equally or more favourably compared to other colleagues [30]. However, it is important to acknowledge the complex relationship between patient satisfaction and quality of care. Patient satisfaction may reflect whether their expectations are met, but not always the actual quality of care. Even though patient experiences and satisfaction are of the utmost importance, patients may report dissatisfaction despite receiving high‐quality care or conversely may express satisfaction even when care is suboptimal [31]. This complexity underscores the need to understand quality care from various views, integrating both subjective experiences and objective measures to achieve improvements in healthcare delivery and person‐centred care. This further strengthens the patient's unique characteristics, resources, needs and life contexts [32, 33], while promoting shared responsibility and ensuring the patient feels heard, seen and valued [34].

The findings indicate that age, sex and educational level did not have a significant impact on patient expectations or experiences of RNs' communication. This contrasts with earlier research suggesting that the sex of both patients and RNs may influence emotional communication. For example, older female patients are often more expressive of emotional concerns, and female RNs are more likely to hear such concerns than their male colleagues [11]. These differences highlight the complexity of communication dynamics and suggest that while demographic factors like age and sex may not directly affect overall satisfaction, they could still play a role in specific aspects of emotional interaction during consultations. Understanding these nuances can help tailor communication approaches to meet diverse patient needs.

Native language was found to be associated with the affect‐oriented aspects of communication. However, determining whether this association is related to native language competence or cultural differences remains challenging. Language and cultural differences can create barriers to healthcare access, interpersonal relationships and outcomes [35]. Furthermore, research shows that patients with different native languages tend to ask fewer questions and are less engaged in the decision‐making process [36]. Schouten et al. [37] highlight the need for culturally sensitive communication, with clear guidance and composure, while Dimond‐Fox and Bone [38] emphasise addressing language barriers, cultural contexts and health literacy as central to all aspects of clinical practice. Gaining insight into communication barriers, particularly those related to language and culture, requires further investigation to support the development of effective communication strategies for patients who are not native speakers.

The results indicate that longer consultations were associated with higher patient satisfaction on both task‐oriented and affect‐oriented scales at 2 weeks follow‐up (T2), consistent with previous findings linking consultation length to satisfaction [39]. However, research suggests that the quality of interaction may outweigh the duration, with effective use of consultation time being critical [29, 40]. Frost et al. [41] highlight that addressing patients' key concerns is essential for satisfaction. While longer consultations may support patient safety and clinical effectiveness [42, 43], studies by Elmore et al. [44] and Swanson et al. [45] found no significant differences in health outcomes based on consultation length. However, it is essential to consider whether the length of the consultation might influence patient satisfaction over time. Further research is required to develop a more comprehensive understanding of this relationship.

### 4.1 Strengths and Limitations

There are some strengths that should be highlighted in the current study. The data was collected from different primary care centres that attend to different populations in an urban area. Measurements were taken at three distinct time points, giving patients the opportunity to evaluate their care after some time had passed. Moreover, the results have been discussed with a reference group comprising representatives of patients and RNs.

There are some limitations that should be considered when interpreting the results. Even though participants were recruited consecutively, this may still have resulted in a certain selection bias. Nonetheless, the distribution of patients in the study was diverse in relation to the patients' gender, age and native language, which has a positive impact on the generalisability of the study. Another limitation in the study is the potential confounding resulting from the lack of randomisation. To minimise this risk, multivariate regression analyses were undertaken and the effect of several variables (e.g., age, education, etc.) was controlled for.

Patients who answered the questionnaire generally reported positive experiences. However, the main reason for nonresponse was a lack of time before consultations. The high satisfaction levels could be influenced by the fact that all participants made an appointment with an RN at the primary care centre, whereas those who were advised on the telephone to manage their condition with self‐care or wait‐and‐see were not included in this study. One of the scales of the QUOTE‐COMM instrument, the affect‐oriented scale, had low internal consistency (Cronbach's *α*), while the rest of the questionnaire demonstrated acceptable reliability. This may be due to the limited number of questions in this scale [46]. Previous studies have shown some variation in Cronbach's *α* values [17, 23]. Due to this lower internal consistency, the results should be interpreted with caution. Future research should consider refining this scale. Although the questionnaire used in this study has been applied in primary care settings before [23, 47], it was originally developed to assess physicians' communication during consultations. The RN‐led consultations in this study were considered comparable to physician consultations in terms of context and communication focus, supporting the questionnaire's relevance in this context. However, the therapy‐oriented nature of the scale posed challenges for some patients, making certain questions more difficult to answer. In some cases, patients found it difficult to distinguish whether specific tasks had been performed by the RN or the physician, which led some to refrain from answering, which may have contributed to a lower response rate. Future research should further examine the applicability of this instrument in an RN context to ensure its validity across different countries and healthcare provider roles.

### 4.2 Practical Implications

#### 4.2.1 Emphasise Communication Training

Given patients' high expectations and the ability of RNs to meet them, ongoing training in effective communication skills should be prioritised to maintain and further enhance these skills.

#### 4.2.2 Promote Person‐Centred Care

Encourage RNs to continue building trust and rapport by focusing on friendliness and attentiveness and addressing patient concerns, as these are key to meeting communication expectations.

#### 4.2.3 Tailored Interventions for Diverse Populations

More research is needed to develop knowledge and strategies to bridge communication gaps with patients from different linguistic or cultural backgrounds to ensure equity in meeting expectations.

## 5 Conclusions

In this study, patients reported high expectations for RN communication in primary care consultations, and RNs seemed to meet their expectations. However, further research is needed to explore the content and goals of patient–RN communication to better understand its moderating and mediating role in meeting patient needs and improving patient outcomes.

## Author Contributions

**Sofia Östensson:** methodology, investigation, writing – original draft, formal analysis, writing – review and editing, data curation. **Mariela Acuña Mora:** conceptualisation, methodology, writing – review and editing, investigation, formal analysis, writing – original draft. **Laura Darcy:** writing – review and editing. **Lotta Saarnio Huttu:** writing – review and editing. **Sandra van Dulmen:** writing – review and editing, conceptualisation. **Annelie J. Sundler:** conceptualisation, methodology, investigation, writing – original draft, writing – review and editing.

## Ethics Statement

Ethical approval was obtained from the Swedish Ethical Review Authority (Dnr Ö24‐2023/3.1).

## Consent

Patients who were willing to participate gave their informed consent.

## Conflicts of Interest

The authors declare no conflicts of interest.

## Data Availability

The data that support the findings of this study are available upon request from the corresponding author. The data are not publicly available due to privacy or ethical restrictions.
